# Transient Receptor Potential Canonical (TRPC) Channels as Modulators of Migration and Invasion

**DOI:** 10.3390/ijms21051739

**Published:** 2020-03-03

**Authors:** Muhammad Yasir Asghar, Kid Törnquist

**Affiliations:** 1 Minerva Foundation Institute for Medical Research, Biomedicum Helsinki 2U, Tukholmankatu 8, 00290 Helsinki, Finland; yasir.asghar@helsinki.fi; 2 Faculty of Science and Engineering, Cell Biology, Åbo Akademi University, Tykistökatu 6A, 20520 Turku, Finland

**Keywords:** TRPC, ion channels, cancer, thyroid, calcium, migration, invasion, angiogenesis

## Abstract

Calcium (Ca^2+^) is perhaps the most versatile signaling molecule in cells. Ca^2+^ regulates a large number of key events in cells, ranging from gene transcription, motility, and contraction, to energy production and channel gating. To accomplish all these different functions, a multitude of channels, pumps, and transporters are necessary. A group of channels participating in these processes is the transient receptor potential (TRP) family of cation channels. These channels are divided into 29 subfamilies, and are differentially expressed in man, rodents, worms, and flies. One of these subfamilies is the transient receptor potential canonical (TRPC) family of channels. This ion channel family comprises of seven isoforms, labeled TRPC1–7. In man, six functional forms are expressed (TRPC1, TRPC3–7), whereas TRPC2 is a pseudogene; thus, not functionally expressed. In this review, we will describe the importance of the TRPC channels and their interacting molecular partners in the etiology of cancer, particularly in regard to regulating migration and invasion.

## 1. Introduction

Increasing evidence during the past decade indicates that different ion channels are expressed in several cancers in humans, and regulate a multitude of cellular processes, including migration, invasion and proliferation [1,2,3]. Hence, ion channels have emerged as potential targets for cancer therapy. Of the channels considered important for cancer progression, special interest has been given to Ca^2+^ channels, as Ca^2+^ signaling is of crucial importance in the regulation of many cellular processes. Different Ca^2+^ channels gate Ca^2+^ ions into the cell, where after Ca^2+^ bind to Ca^2+^ binding proteins and activate downstream signaling pathways, resulting in specific cellular responses. These include motility and contraction, energy production, and gene transcription. To make this versatility possible, several different, pumps, transporters, and channels are necessary [4,5,6].

In many cancer forms, Ca^2+^ signaling plays an essential role in cell migration, invasion, and proliferation through transient receptor potential canonical (TRPC) channels, a subfamily of the transient receptor potential (TRP) superfamily of Ca^2+^ ion channels. The TRPC subfamily comprises six members in humans, TRPC1 and TRPC3–7. Many of these channels are ubiquitously expressed in human tissues and modulate a multitude of cellular responses [7,8,9,10,11,12]. Some of these channels function both as receptor operated Ca^2+^ entry (ROCE) and as store operated Ca^2+^ entry (SOCE) channels [1,13,14]. In the present review, we want to highlight the importance of different TRPC channels in the migration and invasion of cancer cells. For a recent, comprehensive overview of the importance of different ion channels, and not exclusively TRPC channels, see [2,3].

## 2. Calcium Signaling

Ca^2+^ is a ubiquitous intracellular second messenger and activates numerous cellular processes, including fluid secretion, muscle contraction, exocytosis, gene transcription, fertilization, cell differentiation, proliferation and migration [4,15,16]. 

**Figure 1:**
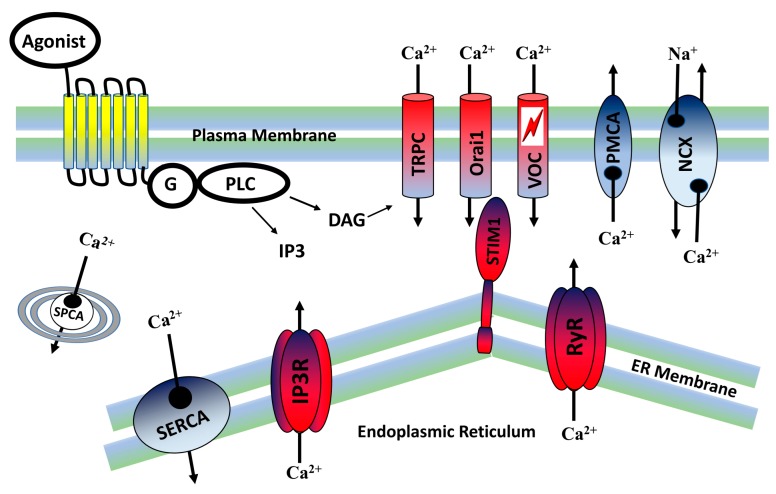
Mechanisms of Ca^2+^ signaling. Upon activation of a G-protein coupled receptor (GPCR) by an agonist, phospholipase C (PLC) is activated, which generates two second messengers; diacylglycerol (DAG) and inositol 1,4,5-triphosphate (IP3). DAG is capable of activating TRPC channels in the plasma membrane and Ca^2+^ influx is triggered. IP3 diffuses through the cytoplasm and binds to IP3 receptors on the endoplasmic reticulum (ER) membranes. This binding enables ER depletion, resulting in a rapid Ca^2+^ transient. The depletion of ER is sensed by stromal interaction protein 1 (STIM1) proteins which act as sensors. STIM1 makes a complex with Orai1 channels in the plasma membrane and induces store operated Ca^2+^ entry through Orai1. Voltage-operated calcium channels open in response to a depolarization of the plasma membrane in excitable neural and muscle cells. Ca^2+^ in the cytoplasm activates ryanodine receptors and Ca^2+^ is released from the endoplasmic reticulum (ER). To avoid a Ca^2+^ flood in the cytoplasm, the pumps sarcoplasmic-endoplasmic reticulum Ca^2+^ATPase (SERCA) and plasma membrane Ca^2+^ATPase (PMCA), and the sodium calcium exchangers (NCX), are activated which export Ca^2+^ out of the cell or into the ER. The secretory pathway Ca^2+^ ATPase (SPCA) transports Ca^2+^ ions into the Golgi apparatus.

Ca^2+^ entry into the cells can be evoked by depolarization of the plasma membrane and the activation of voltage-operated calcium channels (VOCs). These channels are mainly found in excitable cells, such as neurons and muscle cells. An increase in calcium entry can also be obtained in response to an agonist stimulus that, through G protein coupled receptors (GPCR), activates membrane-associated phospholipase C (PLC), and generates two important second messengers, inositol 1,4,5-triphosphate (IP3) and diacylglycerol (DAG). DAG has been shown to activate receptor-operated calcium entry (ROCE) by activation of TRPC channels, which enables Ca^2+^ influx into the cells. The IP3 diffuses into the cytoplasm and activates IP3 receptors on the endoplasmic reticulum (ER), resulting in the release of Ca^2+^ from the ER stores [4,17,18,19,20]. The extracellular Ca^2+^ concentration, which usually is 2–3 mM, is huge as compared to the intracellular free Ca^2+^ concentration at resting state. The resting level of free Ca^2+^ in the cytoplasm is maintained at low concentration, about 100 nM. The steep concentration gradient facilitates rapid Ca^2+^ entry into the cells. Cells maintain the low level of cytosolic Ca^2+^ by strict regulation of Ca^2+^ channels, pumps, and Ca^2+^ binding proteins (Figure 1). The Ca^2+^ signals in cells are normally in the form of rapid transients in the free cytosolic Ca^2+^ concentrations. The abnormality or impairment of the cytosolic Ca^2+^ transients may induce severe diseases [6,20,21].

The increase in the cytoplasmic Ca^2+^ levels enables Ca^2+^ binding proteins such as EF-hand proteins, annexins, and C2-domain proteins to become active and to regulate several cellular processes, such as muscle contraction, exocytosis, metabolism, gene transcription, fertilization, proliferation, and hypertrophy. To avoid too much of Ca^2+^ in the cytoplasm, the levels of Ca^2+^ in the cytoplasm are strictly regulated. However, the Ca^2+^ signals may have a duration ranging from microseconds to hours, depending upon the cell type and the specific function of the signal. Persistently elevated Ca^2+^ levels in the cytoplasm can lead to apoptosis and cell death. Moreover, irregular, e.g., high, or low amplitude Ca^2+^ signals have been associated with diseases [6,21].

## 3. Transient Receptor Potential (TRP) Channels

In the fly, Drosophila melanogaster, a mutant that caused temporary blindness in bright light stimulus was detected, and later it was found that this mutation encodes a cation transient receptor potential (TRP) channel [22,23,24]. The TRP channel-superfamily is the largest family of cation channels and is comprised of 17 different channels in worms, 13 in flies, 28 in mice, and 27 in humans, as shown in Table 1. 

**Figure 2:**
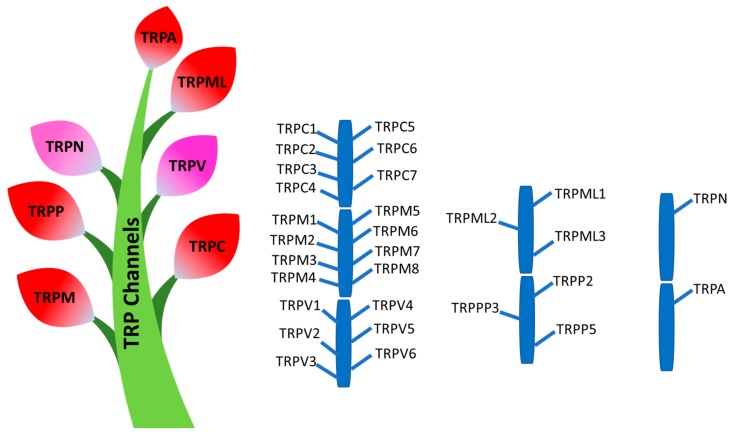
The human phylogenetic tree of the TRP channel superfamily. TRPC (canonical), TRPV (Vanilloid), TRPML (Mucolipin), TRPP (Polycystin), TRPM (Melastatin), TRPA (Ankyrin), and TRPN (NOMPC). TRPC2 is a pseudogene in human. TRPN is expressed in fish.

The expression and function of TRP channels is diverse throughout the animal kingdom, including worms, flies, mice, and humans. There are seven subfamilies of TRP channels based on amino acid sequence homology, and include TRPC (canonical), TRPV (Vanilloid), TRPML (Mucolipin), TRPP (Polycystin), TRPM (Melastatin), TRPA (Ankyrin), and TRPN (no mechanoreceptor potential C), as shown in Figure 2.

TRP channels are selective to cations and some subtypes are highly selective for Ca^2+^ and Mg^2+^. The channels are ubiquitously expressed in human tissues. Many TRP channels have been indicated to participate in a multitude of physiological processes including perception of heat, touch, pain, odor and smell, cellular regulation of osmolarity, fluid secretion, inflammation, cell adhesion, proliferation, cell differentiation, migration, and apoptosis [15,25,26,27,28,29,30,31,32,33,34]. In addition to the physiological importance, TRP channels have been found to regulate many diseases, including cancer [35,36,37,38,39]. 

### 3.1. TRPC Channels

**Figure 3:**
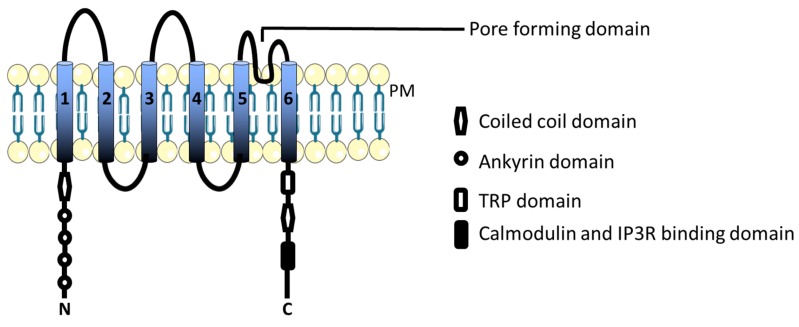
TRPC channel transmembrane structure and the domain organization of the subunits.

The transient receptor potential canonical (TRPC) subfamily consists of seven members (TRPC1-7). In humans, all isoforms are expressed except TRPC2, which is a pseudogene. TRPCs are non-selective cation channels, with a preference for Ca^2+^ over Na^+^ and K^+^ ions. The structure of the TRPC channels consists of six transmembrane segments (S1–S6) connected through loops, and the amino (N) and the carboxyl (C) terminals are located in the cytoplasm. The length and amino acid sequence of the C or N terminals varies. The loop region between segments 5 and 6 makes the pore, which conducts ions as shown in Figure 3. The N-terminus has a coiled-coil domain and four ankyrin domains, which facilitates protein interactions and has been shown to be involved in the regulation of TRPC channel function by tetramerization of TRPC subunits [40]. The C-terminus has a TRP domain, which is the site for other TRP channel isoforms to bind and form channel complexes, a coiled-coil domain, and a calmodulin and IP3R binding site, which regulates the activation and inhibition of the channel [41]. TRPCs regulate several Ca^2+^ dependent cellular processes. TRPC1 can co-assemble with all other TRPC isoforms (TRPC3-7). The TRPC1/TRPC3 complex enhances SOCE, and regulates the differentiation of e.g., H19-7 hippocampal neuronal cells [42]. TRPC channels participate in both ROCE as well as SOCE together with Orai1 and STIM1 proteins.

#### 3.1.1. TRPC1

TRPC1 channels are ubiquitously expressed in human tissues and participate in carrying out many cellular processes, including cell migration and proliferation [11,13,14,43]. TRPC1 is the first mammalian TRPC channel identified and cloned [44]. TRPC1 has been established as a potent molecular component of SOCE in several distinct cell types [19,45,46,47]. In addition, TRPC1 has been shown to function as a ROCE channel in several cell lines [48,49]. TRPC1 can make complexes with all other TRPC channels. In HSY cells, TRPC1 forms a complex with TRPC3. In human embryonic kidney (HEK-293) cells, TRPC1 forms a heterotrimeric complex with both TRPC3 and TRPC7. In mesangial cells, TRPC1 forms a complex with TRPC4 and in neuronal cells TRPC1 has been shown in complex with TRPC5 [11,50,51,52,53]. TRPC1 channels also interact with the Ca^2+^ signaling proteins G_q/11_, PLC, calmodulin, IP3 receptors (IP3R), PMCA, SERCA, and STIM1 to regulate cellular processes. STIM1 and the TRPC1-TRPC4 complex are essential for store refilling and differentiation in myotubes [54].

#### 3.1.2. TRPC2

TRPC2 is a pseudogene and does not form a functional channel in humans [44,55]. Relatively little is known about this channel’s physiology and pathology. However, in other mammals, TRPC2 forms a functional channel in distinct tissues, such as the vomeronasal organ (VNO), testis, spleen and liver [55,56]. The importance of TRPC2 in the vomeronasal organ has obtained much interest in regard to pheromone detection, see reviews [57,58]. TRPC2 has been shown to interact with Homer 1, calmodulin, G_q/11_, IP3R, and receptor transporting protein 1 (RTP1) [59,60,61]. We detected TRPC2 in the rat thyroid FRTL-5 cell line. Knockdown of TRPC2, or expression of a dominant negative form of TRPC2 in these cells resulted in a significant decrease in proliferation, migration, adhesion and invasion [62]. In addition, TRPC2 has the ability to mediate both ROCE and SOCE in FRTL-5 cells [63].

#### 3.1.3. TRPC3

TRPC3 channels are expressed in the human brain, kidney, skeletal muscle, mammary, ovary and cardiovascular tissues [14]. TRPC3 interacts and forms heterodimers with TRPC1, 4, 5, 6, and 7. Despite a considerable homology of the amino acid sequence between TRPC3, TRPC6, and TRPC7, these channels regulate many different functions in humans [64,65]. TRPC3 also interacts with the Orai1 calcium channel [66,67]. TRPC3, either in complex with TRPC1, or in complex with both TRPC1 and TRPC7, forms a SOCE channel. TRPC3 interacts with several signaling proteins in response to receptor-evoked Ca^2+^ mobilization, including PLCβ, G_q/11_, and IP3R. TRPC3 has been reported to be directly activated by DAG [68].

#### 3.1.4. TRPC4

TRPC4 shares a 65% homology in the amino acid sequence with TRPC5 [19,45]. Mostly, TRPC4 channels form SOCE channels, but in some cell types, they form store-independent channels as well. It can make heteromeric channels with TRPC6. STIM1 binds with TRPC4 and regulates its function as a SOCE channel [69]. Furthermore, TRPC4 has been studied extensively in regard to endothelial cell function and has been shown to regulate proliferation of these cells [14,70].

#### 3.1.5. TRPC5

TRPC5 is highly expressed in neuronal cells and regulates their function. In HEK-293 cells, TRPC5 is a non-selective channel and functions as a ROCE channel but not as a SOCE channel [71]. Recently, it has been shown that TRPC5 is activated in response to mechanical stress, and this increase in the activity of the channel is dependent on actin filaments [72]. TRPC5-TRPC1 complexes are found in neurons, vascular endothelial cells, and smooth muscle cells. In vascular smooth muscle (VSM) cells, TRPC5 has been suggested to act as SOCE channel by forming complexes with TRPC1, TRPC6, and TRPC7 [73].

#### 3.1.6. TRPC6

TRPC6 is expressed in pulmonary and vascular smooth muscle cells. These channels are directly activated by DAG and do regulate ROCE [74]. However, activation of TRPC6 due to Ca^2+^ release from the ER is mediated by Orai1 and TRPC4 [75]. TRPC6 has been shown to make a complex with TRPC3 in neuronal cells and prostate cancer epithelial cells. In astrocytes, sphingosine 1-phosphate (S1P)-evoked secretion of the C-X-C motif chemokine ligand 1 (CXCL1) is mediated through activation of TRPC6 and the mitogen-activated protein kinase (MAPK) signaling pathway [76]. Furthermore, TRPC6 is important in transforming growth factor β1 (TGFβ1) signaling in vascular smooth muscles. TGFβ1 induces stress fiber formation in these cells via upregulation of TRPC6 [77]. The interplay of leukocytes with platelet/endothelial cell adhesion molecule-1 (PECAM) result in activation of TRPC6 which modulates subsequent leukocyte transendothelial migration (TEM) [78].

#### 3.1.7. TRPC7

In humans, TRPC7 is widely expressed in many tissues including the brain, skin, cartilage, pituitary gland, intestine, kidney, and prostate [14]. TRPC7, a non-selective cation channel, is the seventh identified member of mammalian TRPC family. It was isolated through molecular cloning of the mouse fetal brain and caudate nucleus cDNA libraries [79,80]. The role of TRPC7 in the regulation of normal cell physiology and pathology is still indefinable. TRPC7 is activated by G_q_-coupled protein receptors and the PLC pathway and is directly activated by DAG. In some cell types, TRPC7s are constitutively active proteins and may function as SOCE channels by forming a TRPC1-TRPC3-TRPC7 complex. However, in HEK-293 cells, a TRPC3 and TRPC7 complex is activated by DAG and functions as ROCE channels [81]. Furthermore, cGMP-dependent protein kinase 1α, calmodulin, IP3R, and phosphatidylinositol 4,5,-bisphosphate (PIP2) have all been reported to regulate the function of TRPC7 [82]. In addition, activation of TRPC7 potently induces myocardial apoptosis [83].

## 4. TRPC Channels as Regulators of Migration and Invasion

### 4.1. TRPC Channels, Migration and Invasion

Several TRPC channels are involved in regulating migration and invasion, and all are involved in enhancing proliferation of cancer cells [84,85,86]. TRPC1 channels are essential for the polarity and direction of migrating cells both in vitro and in vivo [49,51,52]. The directionality of migrating cells, as shown using renal transformed epithelial cells, was dependent on TRPC1 [87]. In aggressive glioma cells, TRPC1 regulates epidermal growth factor (EGF)-evoked migration [88]. In addition, TRPC1 has been shown to localize to lipid rafts at the leading edge of migrating cells. TRPC1 mediated Ca^2+^ entry has been shown to activate the MAPK and phosphoinositide 3-kinase/Akt (PI3K/Akt) signaling pathways, calpains, hypoxia-induced factor 1α (HIF1α) and several matrix-metalloproteinases (MMPs). Thus, TRPC1 has emerged as an important player involved both in normal and cancer cell function [49]. Furthermore, TRPC1 may be in complex with the small conductance Ca^2+^ activated potassium channel (SK3) and Orai1 to enhance SOCE-dependent colon cancer migration [89]. In skeletal myoblast migration, TRPC1 is of importance to evoke Ca^2+^ -mediated activation of calpains [11]. Silencing TRPC1 by siRNA inhibited invasion of CNE2 nasopharyngeal tumor cells [90].

We have shown that TRPC1 is a potent regulator of both migration and invasion in follicular thyroid cancer ML-1 cells [91]. The mechanisms involved appear to depend on the at least MAPK/ERK1/2, MMP2 and-9, and HIF1α. However, so far, no studies regarding TRPC1 in thyroid cancer have been performed. The importance of TRPC channels in normal thyroid cells has, to the best of our knowledge, not been investigated.

The importance of TRPC2 as a regulator of invasion and migration has not been extensively studied. In thyroid cells, Ca^2+^ has many important functions [63,92], and we have shown that, in normal rat thyroid FRTL-5 cells, TRPC2 potently regulates migration, invasion, and adhesion [62]. Rac, calpain and MMP2 seem to be of importance in TRPC2-regulated migration. Considering that thyroid cells migrate during embryogenesis [93], our data suggest that TRPC2 might have a role in this event in rodents. Although TRPC2 knock-out mice appear normal (except for their behavioral changes, see e.g., [94]), it would also be of interest to investigate the thyroid status in these mice. 

The importance of TRPC3 as a regulator of migration and invasion has been studied in several different tumor cells. In human ovarian cancer, the expression of TRPC3 was increased, and injection of TRPC3-knock down SKOV3 cells decreased tumor formation in nude mice [95]. In pancreatic stellar cells, activation of KCa3.1 results in activation of TRPC3-evoked Ca^2+^ entry, calpain activity, and concomitant cell migration [96]. TRPC3 also enhanced melanoma cell migration and tumor formation both in vitro and in vivo [97]. Furthermore, TRPC3, together with mGluR5, has been shown to enhance motility in embryonic neuronal cells as a result of endocannabinoid stimulation [98]. It has also been shown that the invasion of bladder cancer cells is dependent on both TRPC3 and TRPC6. Interestingly, in these cells the expressional level of these channels is under the control of the histone variant macroH2A [99].

Not much is known regarding the importance of TRPC4 or TRPC5 in enhancing migration and invasion of cancer cells, although at least TRPC4 seems to have a role in proliferation and tumor formation [100]. Furthermore, TRPC4 seems to enhance invasion of certain forms of medulloblastoma cells. In these cells, the expression of TRPC4 appears to be regulated by the proton sensing G-protein coupled receptor 1 (OGR1) [101]. Several TRPC isoforms, including TRPC4, have also been suggested to enhance proliferation of non-small cell lung cancer [102]. The importance of TRPC5 has been shown in regulating hippocampal neurite length and growth cone morphology, by having an inhibiting role in neurite extension [103]. Furthermore, in fibroblasts and kidney podocytes, TRPC5 is in complex with Rac1, enhancing cell motility, whereas TRPC6-evoked Ca^2+^ entry enhances RhoA activity; thus, inhibiting cell motility [104]. Interestingly, the plant derived compound Englerin A induces cytotoxicity in some cancer cell lines expressing TRPC4 and TRPC5 by enhancing channel activity; thus, increasing cytosolic Ca^2+^ and so Na^+^ concentrations [105,106].

In addition to TRPC1 and TRPC3, the involvement of TRPC6 in cancer cell invasion and migration has been extensively investigated [3]. In human head and neck squamous cell carcinomas (HNSCC), TRPC6 was overexpressed, and knock-down of TRPC6 in HNSCC potently attenuated invasion [107]. TRPC6 was also shown to be of significant importance in regulating Notch-driven glioblastoma growth and invasiveness [108]. In human prostate cancer epithelial (hPCE) cells, active TRPC6 and nuclear factor of activate T cells (NFAT) promote proliferation via alpha 1-adreniergic receptor signaling [109]. In non-small cell lung cancer A549, TRPC6 was important for invasion [110]. Interestingly, knock-down of TRPC6 not only attenuated invasion, but also decreased the expression of the adhesion protein fibronectin and the tight junction protein ZO-1 [110]. Furthermore, TRPC6 was of importance in regulating breast cancer MDA-MB-231 cell migration [111]. TRPC6 expression has been found upregulated in breast cancer cell lines, MCF-7 and MDA-MB-231, compared with normal breast epithelial MCF10A cells. In vitro knockdown of TRPC6 decreased proliferation and migration in these cancer cells. In addition, TRPC6 has been shown to interact and translocate Orai1 and Orai3 to the plasma membrane upon ER Ca^2+^ store depletion [112].

An overview of TRPC channels expressed in tissues and their respective reported function regarding invasion and migration has been summarized in Table 2.

### 4.2. TRPC Channels and Angiogenesis

The regulation of angiogenesis is a complex event where calcium signaling plays a significant role. Endothelial cells express several members of the TRP superfamily, many of which have been shown to be involved in angiogenesis. Knockdown or inhibition of TRPC3 concealed the endothelial tube formation by attenuation of VEGF-evoked Ca^2+^ entry and phosphorylation of MAPK [113], and inhibition of TRPC4 suppressed the VEGF evoked neovascularization in human retina microvascular endothelial cells (HRMECs) [114]. TRPC3, 4 and 5 have been shown to participate in in vitro tube formation of the human umbilical vein endothelial cell-derived cell line EA.hy926 [115], and in mice with ischemic injury, the knockdown of TRPC5 in endothelial cells (ECs) under-hypoxia inhibited sprouting and tube formation. However, activation of TRPC5 in these cells restored angiogenesis [116]. TRPC6 has been shown to participate in VEGF evoked sprouting and angiogenesis in human microvascular endothelial cells (HMVEC) [117]. Furthermore, phosphatase and tensin homolog (PTEN) regulates TRPC6 activation and subsequent promotion of angiogenesis in primary human pulmonary artery endothelial cells (HPAEs) [118]. As several comprehensive reviews regarding TRP channels and angiogenesis have recently been published, we recommend the readers to turn to these for further information [119,120,121].

## 5. Conclusions

It seems clear that TRPC channels (and apparently, also, other members of the TRP superfamily), can have a profound role in the migration and invasion of cancer cells, and thus in the process of metastasis. Furthermore, as pointed out by a recent review from the Prevarskaya lab, in many of the common cancer hallmarks, i.e., uncontrolled proliferation, resistance to programmed cell death, invasion and metastasis, and angiogenesis, ion channels (including TRP channels) have a clear role [3]. Thus, it seems obvious that different ion channels are considered putative targets for pharmacological interventions. However, as the TRPC channels often are ubiquitously expressed, it will be a challenge to find suitable pharmacological tools to block these channels and curtail cancer without severe side effects. One possibility could be functionalized nanoparticles that specifically target cancer cells, see e.g., [122,123]. This area of research is proceeding rapidly, and novel approaches are constantly developed. It will be interesting to see if these will be used to e.g., carry siRNA to target ion channels in cancer cells, and whether this approach can be used in patients.

## Figures and Tables

**Table 1:** Expression of transient receptor potential (TRP) channels in worms, flies, mice, and humans.

	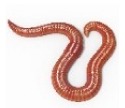	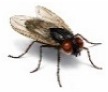	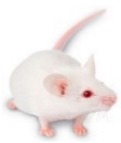	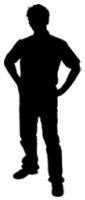
**TRPC**	3	3	7	6
**TRPV**	5	2	6	6
**TR**PM	4	1	8	8
**TRPA**	2	4	1	1
**TRPML**	1	1	3	3
**TRPP**	1	1	3	3
**TRPN**	1	1	0	0
**Total**	17	13	28	27

**Table 2:** Effects of TRPC channels on invasion, migration, and proliferation.

Channel	Tissue	Cell Line	Cell function	References
TRPC1	Kidney	MDCK-F	Migration	[87]
	Brain cancer	D54MG	Migration	[88]
	Colon cancer	HCT-116	Migration	[89]
	Skeletal Muscle	C2C12	Migration, differentiation	[11]
	Nasopharyngeal cancer	CNE2	Migration, Invasion	[90]
	Thyroid cancer	ML-1	Migration and Invasion	[91]
TRPC2	Rat Thyroid	FRTL5	Proliferation, adhesion, migration and invasion	[62]
TRPC3	Ovarian cancer	SKOV3	Migration	[95]
	Pancreatic stellate cells	RLT-PSC	Migration	[96]
	Melanoma	C8161	Migration	[97]
	Neuronal progenitor cell	NPCs	Migration	[98]
	Bladder Cancer	LD611	Migration and Invasion	[99]
TRPC4	Coronary artery	HCAECs	Proliferation	[70]
	Ovarian cancer	SKOV3	Proliferation	[100]
	Medulloblastoma	DAOY	Migration, Invasion	[101]
	Lung cancer	A549	Proliferation	[102]
TRPC5	Rat hippocampal neuron	E18	Inhibition of neurite extension	[103]
	Mouse Kidney	Podocytes	Promote migration	[104]
TRPC6	Mouse Kidney	Podocytes	Inhibit migration	[109]
	Head and neck cancer	HNSCC	Promote Invasion	[107]
	Brain cancer	U373MG	Migration, cell growth	[108]
	Prostate cancer	hPCE	Proliferation	[109]
	Lung cancer	A549	Proliferation	[110]
	Breast Cancer	MDA-MB-231	Migration	[111]
